# *Rhamnella intermedia* (Rhamnaceae), a new evergreen species from southwest Guangxi

**DOI:** 10.3897/phytokeys.159.53177

**Published:** 2020-09-04

**Authors:** Zhiqiang Lu, Yongshuai Sun

**Affiliations:** 1 CAS Key Laboratory of Tropical Forest Ecology, Xishuangbanna Tropical Botanical Garden, Chinese Academy of Sciences, Mengla 666303, Yunnan, China Chinese Academy of Sciences Mengla China; 2 Center of Plant Ecology, Core Botanical Gardens, Chinese Academy of Sciences, Mengla 666303, Yunnan, China Chinese Academy of Sciences Mengla China

**Keywords:** evergreen species, independent evolutionary lineage, intermediate morphology, phenotypic cluster, *
Rhamnella
*

## Abstract

*Rhamnella intermedia*, a new evergreen species from southwest Guangxi, is described and illustrated in this study. This species is similar to *R. brachycarpa* by the size and ratio of length to width of dried fruit and seeds, by which it differs from *R. rubrinervis* and *R. tonkinensis*. However, it differs from *R. brachycarpa* by rarely mucronate seed apices, larger ratio of length to width of leaves, leaf apices acuminate to long acuminate, shorter leaf petioles, and longer fruiting pedicels. Principal component analysis based on phenotypic traits further recognised three separated groups. *Rhamnella rubrinervis* and *R. tonkinensis* were clustered into one group; the other two groups represented *R. brachycarpa* and two Guangxi populations, respectively. Furthermore, phylogenetic analysis of nuclear ITS sequence variations highly supported that the two Guangxi populations represented an independent evolutionary lineage and were closest to *R. rubrinervis*. Four fixed nucleotide sites were found and were different from *R. rubrinervis*. However, besides the differentiated traits in seeds and fruit, densely pilose young branches also separated them from *R. rubrinervis*. In addition, during our field investigations, none of the three closely related species were found at locations where this new species was distributed. Therefore, this new species, based on the two Guangxi populations, is named *R. intermedia*. The key to four closely related species is also presented.

## Introduction

*Rhamnella* (Miquel, 1867) in the tribe Rhamneae Hook. f. of Rhamnaceae is a small genus (Hauenschild et al. 2016, Richardson et al. 2000). It is characterised by pedicellate flowers and fleshy fruits, 1-stoned drupes, pinnately veined leaves, serrate leaf margins, semi-inferior ovaries, stipules without thorns, and flowers in axillary cymes (Chen and Schirarend 2007). To date, 10 species have been accepted into this genus (Lu and Sun 2019). Two groups are separated based on the characters of deciduous broad-leaved and evergreen leaves respectively (Fan and Yang 1997; Chen and Schirarend 2007; Lu and Sun 2019). For all species in the latter group, including *R. rubrinervis*, *R. tonkinensis*, and *R. brachycarpa*, the length of leaf petioles, leaf apices, ratio of length to width of leaves, and size and ratio of length to width of dried fruit and seeds are their differentiated traits (Chen and Schirarend 2007; Lu and Sun 2019). However, on the basis of these traits, two *Rhamnella* populations from southwest Guangxi that belonged to the evergreen group could not be ascribed to any of the three evergreen *Rhamnella* species. We found that they had similar size and ratio of dried fruit and seeds to *R. brachycarpa*, similar leaf shapes to *R. rubrinervis*, and the same length of fruiting petioles with *R. tonkinensis*. Therefore, the two Guangxi populations may represent a new species. In order to clarify this hypothesis, we carried out field investigations on the distribution and habitat of this potential new species, and characterised its morphology based on these two populations from southeast Guangxi. Furthermore, we conducted the principal component analysis (PCA) based on phenotypic traits to show their morphological differences. Finally, we sequenced the nuclear internal transcribed spacer (ITS) fragment to clarify its genetic distinctness.

## Material and methods

We collected the fruit-bearing specimens of this potential new species for morphological measurement and other analyses referring to Lu and Sun (2019). These newly collected specimens in this study were deposited as *Z.Q. Lu 2019YG2601*– *Z.Q. Lu 2019YG2619* (GXMI and HITBC), *Z.Q. Lu 2018LZQ108* (HITBC), and *Z.Q. Lu 2018LZQ10802* (HITBC). In order to demonstrate its morphological differences, we compared them to all closely related evergreen *Rhamnella* species that had been shown in Lu and Sun (2019), including *R. rubrinervis*, *R. tonkinensis*, and *R. brachycarpa*. In addition, 21 newly collected and 239 previous specimens were further used to perform the PCA based on 10 phenotypic traits, as described by Lu and Sun (2019). Because they were attributed to evergreen taxa closely related to *R. rubrinervis*, *R. tonkinensis*, and *R. brachycarpa*, and were distinctly different from deciduous species of *Rhamnella* by larger drupe size (Chen and Schirarend 2007; Lu and Sun 2019), we excluded all deciduous species from morphological comparison and PCA analysis. Furthermore, we investigated the population consensus and explored whether other closely related species co-occurred with this potential new species. The habitat and distribution were also characterised through our field investigations.

We also collected fresh leaves from several populations for DNA extraction and sequencing (Table 1). Taking habit differentiation into consideration, we marked each of the climbing trees. In total, 21 individuals (including six climbing trees and 15 erect ones) from the potential new species based on Pingxiang and Wude populations, 21 individuals from 6 populations of *R. rubrinervis*, one individual from *R. tonkinensis*, and one individual from *R. brachycarpa* were used for sequencing the nuclear ITS fragment. PCR amplification was performed according to Lu et al. (2018). In total, 5 of 21 individuals failed in the ITS sequencing, but the six climbing individuals were all sequenced successfully. Finally, we obtained 37 ITS sequences, including 7 types. All were deposited in GenBank database (Accession numbers from MT764159 to MT764165). In addition, we also downloaded some ITS sequences of deciduous species of *Rhamnella*. The aligned sequences were 630 bp in length. A maximum likelihood (ML) tree based on ITS sequences was constructed by MEGA version 5.0 (Tamura et al. 2011) using the Tamura-Nei model, and the bootstrap was set as 1000. *Berchemiella wilsonii*, *B. berchemifolia*, *Pseudoziziphus celata*, and *P. parryi* were selected as outgroups referring to the phylogenetic backbone presented by Hauenschild et al. (2016).

**Table 1.** Voucher number and location of populations of collected leaves used for DNA extraction and sequencing in this study.

Species	Location	Latitude (N) / Longitude (E)	Altitude (m)	No. of individuals	Voucher No.
*R. intermedia* Pop1	Pingxiang, Guangxi	22°07'19"N, 106°44'40"E	298	6	*Z.Q. Lu 2019YG2601– Z.Q. Lu 2019YG2614*
*R. intermedia* Pop2	Wude, Guangxi	22°34'15"N, 106°44'56"E	276	2	*Z.Q. Lu 201810801–Z.Q. Lu 201810802*
*R. rubrinervis* Pop1	Chengxiang, Guangxi	23°28'04"N, 105°54'19"E	1000	3	*Z.Q. Lu 201726501– Z.Q. Lu201726503*
*R. rubrinervis* Pop2	Dongying, Guangxi	23°13'23"N, 105°56'32"E	960	4	*Z.Q. Lu 201802701–Z.Q. Lu 201802704*
*R. rubrinervis* Pop3	Dongjing, Guangxi	23°39'8"N, 106°33'22"E	460	6	*Z.Q. Lu 201818201–Z.Q. Lu 201818206*
*R. rubrinervis* Pop4	Dizhou, Guangxi	22°58'38"N, 106°21'8"E	790	3	*Z.Q. Lu 201819001–Z.Q. Lu 201819003*
*R. rubrinervis* Pop5	Debao, Guangxi	23°26'46"N, 106°29'52"E	830	2	*Z.Q. Lu 201818601–Z.Q. Lu 201818602*
*R. rubrinervis* Pop6	Donggan, Yunnan	23°30'19"N, 105°11'45"E	940	3	*Z.Q. Lu 201811101–Z.Q. Lu 201811103*
*R. tonkinensis*	Maogan, Hainan	18°35'36"N, 109°25'48"E	620	1	*Z.Q. Lu 2018HN3001*
*R. brachycarpa*	Maogan, Hainan	18°35'37"N, 109°25'34"E	650	1	*Z.Q. Lu 2019HN101*

## Results

**Figure 1.**
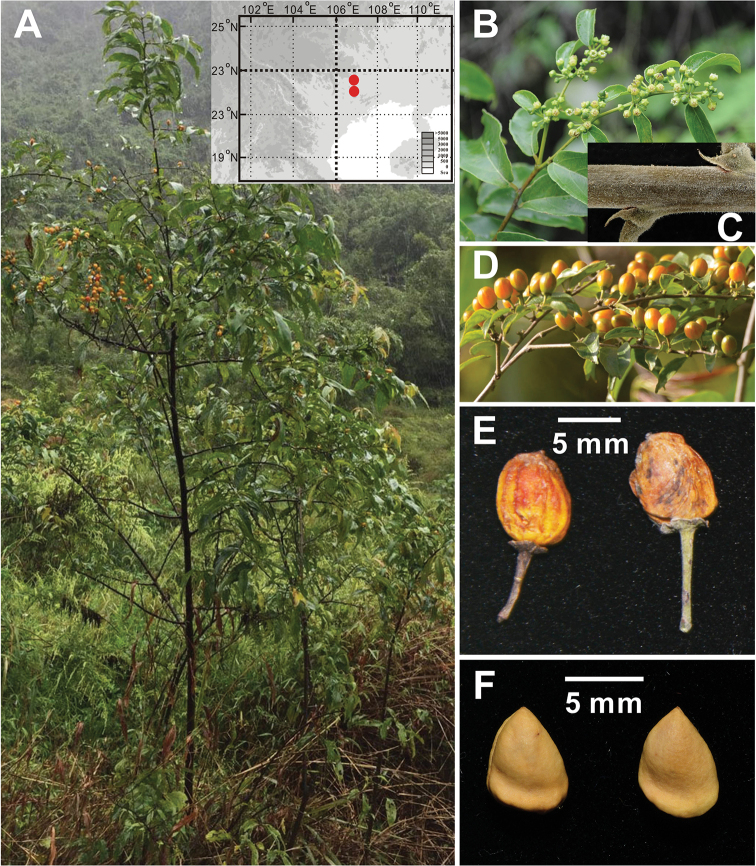
*Rhamnella intermedia* Z. Qiang Lu & Y. Shuai Sun. **A** The whole plant, habitat and two geographical locations **B** flowering branches **C** a young branch with the persistent stipule **D** branches with leaves and fruit **E** dried fruit **F** mature seeds.

**Figure 2.**
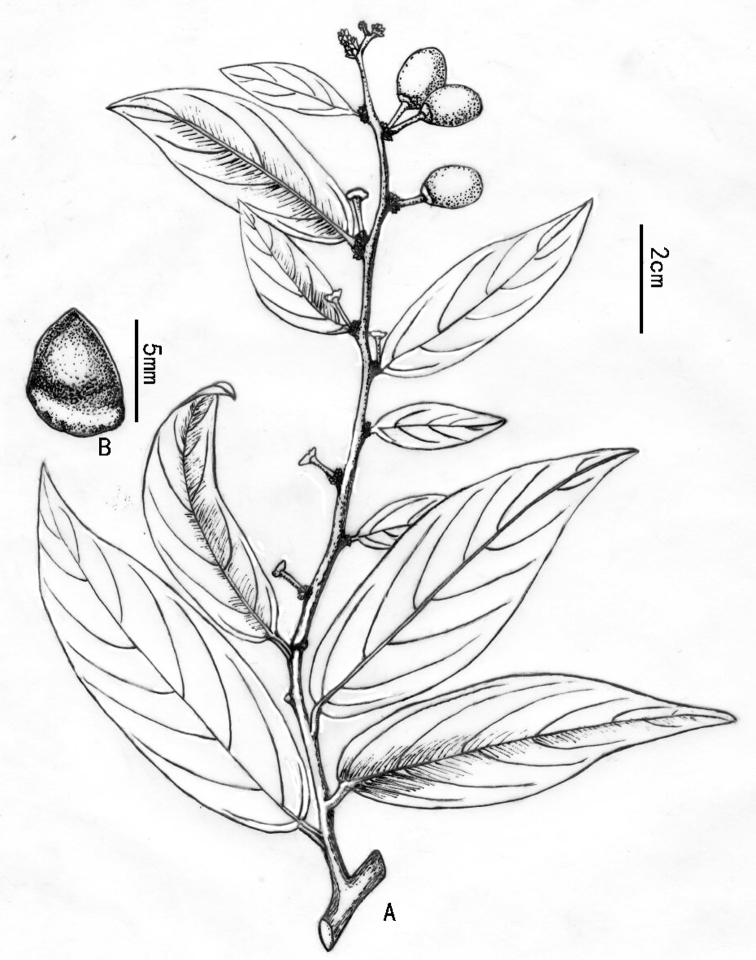
*Rhamnella intermedia* Z. Qiang Lu & Y. Shuai Sun, sp. nov., drawn from *Z.Q. Lu 2019YG2601*.

**Figure 3.**
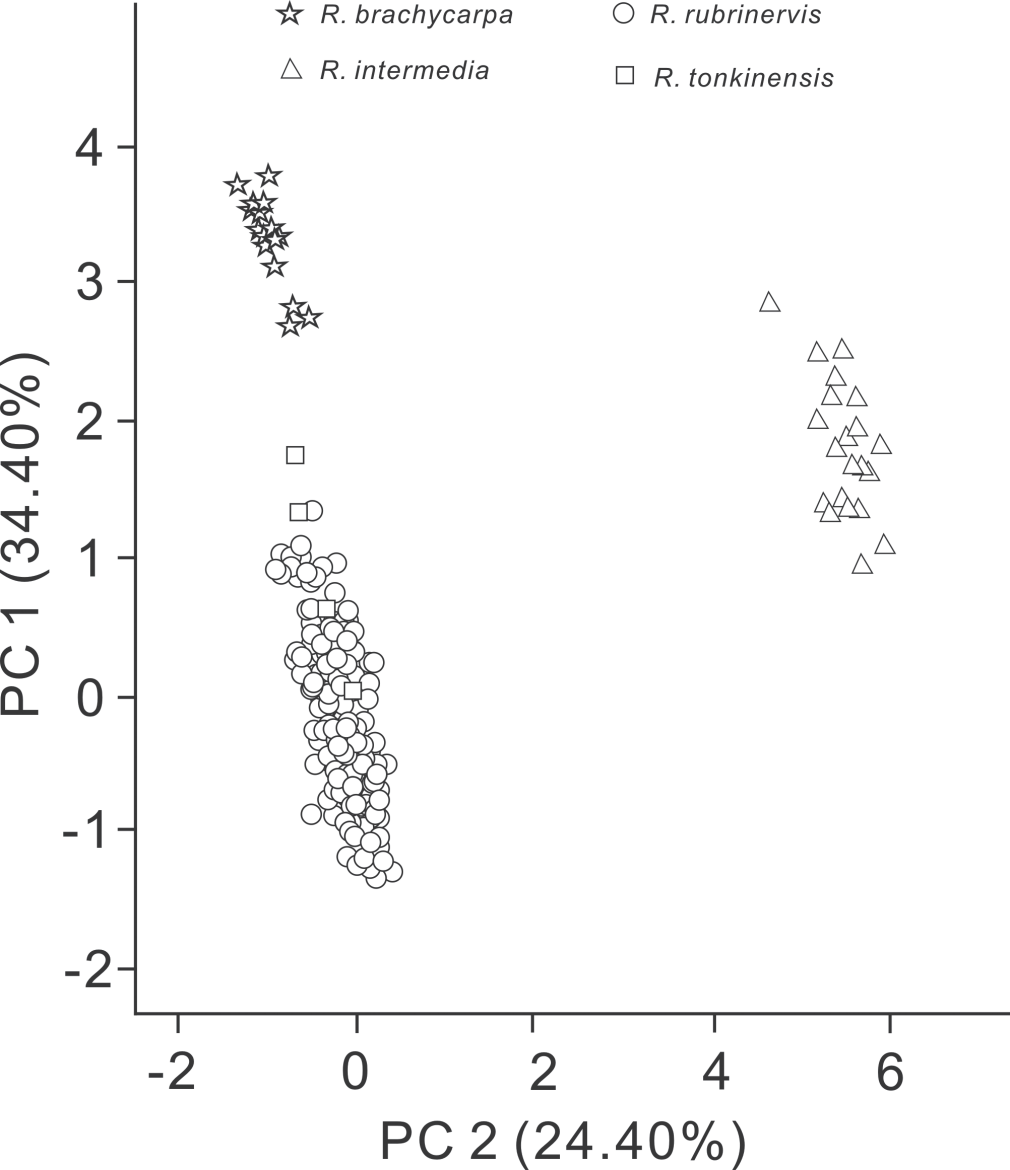
Phenotypic clustering based on principal component analysis.

**Figure 4.**
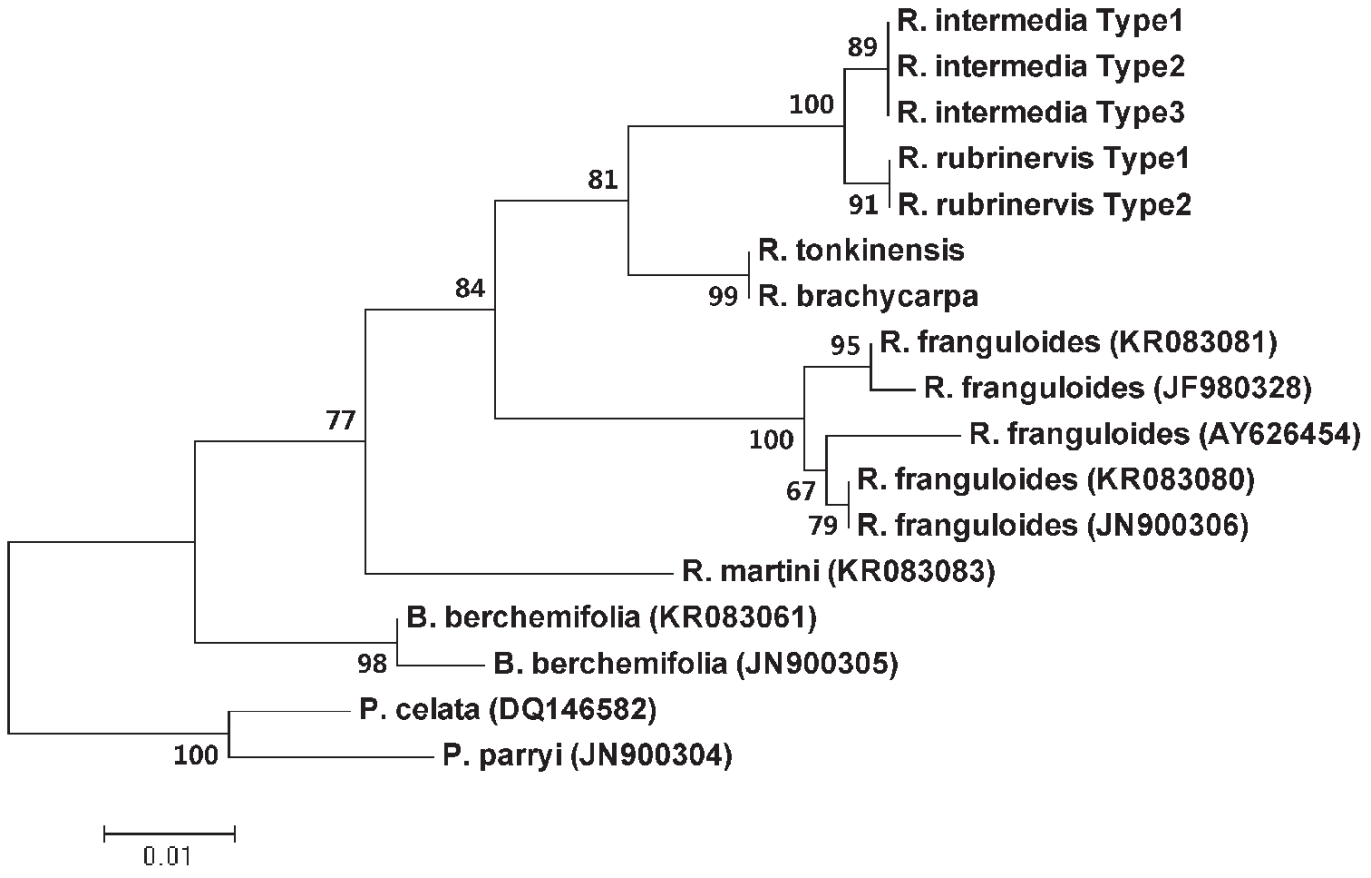
Phylogenetic analysis of evergreen *Rhamnella* species based on nuclear ITS sequences. *Berchemiella wilsonii*, *B. berchemifolia*, *Pseudoziziphus celata*, and *P. parryi* are outgroups.

Two Guangxi populations that are considered as new species have an intermediate morphology compared to the three closely related species *R. rubrinervis*, *R. tonkinensis*, and *R. brachycarpa* (Figures 1, 2; Table 2). Morphological comparison based on the characteristics of fruit and seeds showed that they shared maximum resemblance to *R. brachycarpa* by the similar sizes of dried fruit (6.0–7.0 × 4.7–5.3 vs. 6.5–7.5 × 4.7–6.0 mm) and seeds (5.5–6.5 × 4.5–5.0 vs. 5.0–7.0 × 4.5–5.5 mm), and ratios of length to width of dried fruit (1.3–1.5 vs. 1.3–1.5) and seeds (1.2–1.4 vs. 0.9–1.5). Meanwhile, they differed from *R. brachycarpa* by the larger ratio of length to width of leaves (2.7–3.8 vs. 1.9–2.4), smaller length of leaf petioles (3–8 vs. 7–12 mm), larger length of fruiting pedicels (5–9 vs. 4–6 mm), leaf apices acuminate to long acuminate, and seed apices rarely mucronate. Furthermore, morphological comparison showed that these two Guangxi populations were also morphologically similar to *R. rubrinervis* by the ratio of length to width of leaves (2.7–3.8 vs. 2.7–4.3), leaf apices acuminate to long acuminate, length of leaf petioles (3–8 vs. 3–9 mm) and rarely mucronate seed apices, and to *R. tonkinensis* by the length of fruiting petioles (5–9 vs. 5–9 mm). In contrast, they differed from *R. rubrinervis* and *R. tonkinensis* by smaller sizes of dried fruit (6.0–7.0 × 4.7–5.3 vs. 7.5–11.1 × 4.2–5.8 mm) and seeds (5.5–6.5 × 4.5–5.0 vs. 7.0–9.9 × 4.2–5.5 mm), and smaller ratios of length to width of dried fruit (1.3–1.5 vs. 1.6–2.2) and seeds (1.2–1.4 vs. 1.6–2.1). In addition, they were also separated from *R. rubrinervis* by densely pilose young branches, and from *R. tonkinensis* by the ratio of length to width of leaves (2.7–3.8 vs. 2.1–2.8). In total, two Guangxi populations characterised by the ratio of length to width of leaves and length of leaf petioles from *R. rubrinervis*, by the length of fruiting petioles from *R. tonkinensis*, and by the size and ratio of length to width of dried fruit and seeds from *R. brachycarpa*. Other phenotypic traits, such as leaf apices acuminate to long acuminate and rarely mucronate seed apices also showed a morphological combination from other species. PCA analysis distinguished 260 specimens into three groups, based on 10 phenotypic traits (Figure 3). One group consisted of *R. rubrinervis* and *R. tonkinensis*, while the other two groups corresponded to *R. brachycarpa* and two Guangxi populations respectively. The first principal component axis (PC1; accounting for 34.40% of the variation) significantly separated these *R. brachycarpa* specimens and those of *R. tonkinensis* and *R. rubrinervis* into two clusters, and the two Guangxi populations showed the overlap with both clusters; the second principal component axis (PC2; accounting for 24.40% of the variation) significantly separated the two Guangxi populations from the other two clusters, and failed in the separation of others (Figure 3). In addition, during our field investigations, none of the three closely related species *R. brachycarpa*, *R. tonkinensis*, and *R. rubrinervis* were found at locations where these two Guangxi populations distributed. Furthermore, phylogenetic analysis highly supported that the evergreen group was monophletic, and these two Guangxi populations represented an independent evolutionary lineage distinctly different from other species (Figure 4). They were closest to *R. rubrinervis* based on ITS sequence variations; however, four fixed nucleotide sites were found (Table 3). Sequence alignments showed that six climbing trees shared the same ITS sequence types with those of erect ones.

**Table 2.** Morphological comparision of *Rhamnella intermedia*, *R. rubrinervis*, *R. tonkinensis* and *R. brachycarpa*. Traits that differ between species are marked in bold.

Characters	*R. intermedia*	*R. rubrinervis*	*R. tonkinensis*	*R. brachycarpa*
**LEAF**				
Shape and size	Leaf blade oblong or ovate-oblong, 6.4–13.0 × 2.0–5.0 cm, **length-width ratio 2.7–3.8**; base commonly rounded, rarely cuneate, margin inconspicuously remotely serrate or subentire; **apex acuminate to long acuminate**; bracteole leaf similar to leaves in vegetative branches, but smaller	Leaf blade oblong or ovate-oblong, 5.4–14.4 × 1.7–5.1 cm, **length-width ratio 2.7–4.3**; base commonly rounded, rarely cuneate, margin inconspicuously remotely serrate or subentire; **apex acuminate to long acuminate**; bracteole leaf similar to leaves in vegetative branches, but smaller	Leaf blade elliptic-ovate, 6.5–11.2 × 3.1–4.5 cm, **length-width ratio 2.1–2.8**; base cuneate or nearly rounded, margin inconspicuously remotely serrate or subentire; **apex short acuminate to long acuminate or acute**; bracteole leaf similar to leaves in vegetative branches, but smaller	Leaf blade elliptic-ovate, 5.8–10.3 × 3.1–4.8 cm, **length-width ratio 1.9–2.4**; base cuneate or nearly rounded, margin inconspicuously remotely serrate or subentire; **apex short acuminate or acute**; bracteole leaf similar to leaves in vegetative branches, but smaller
**Length of petiole**	**3–8 mm**	**3–9 mm**	**7–11 mm**	**7–12 mm**
Lateral veins on each side of midvein	6–7	5–8	5–6	5–7
Average distance between lateral veins located in the middle of leaf	3–8 mm	3–8 mm	3–6 mm	3–6 mm
**BRANCH**				
Young branches densely pilose or not	Densely pilose	Sparsely pilose or glabrous	Incompletely clear	Sparsely pilose or glabrous
Stipules lanceolate or subulate	Subulate	Lanceolate or subulate	Incompletely clear	Subulate, but seemingly lanceolate when young
**FLOWER**				
Number of flowers for each axillary cyme	3–10	2–10	3–11	2–9
**Length of pedicel**	4–7 mm	2–5 mm	5–7 mm	3–5 mm
Shape and size	Flower diameter ca. 4 mm; sepals triangular, ca. 2 mm; stamens involute by petals, ca. 2 mm in length	Flower diameter ca. 4 mm; sepals triangular, ca. 2 mm; stamens involute by petals, ca. 2 mm in length	Flower diameter ca. 4 mm; sepals triangular, ca. 2 mm; stamens involute by petals, ca. 2 mm in length	Flower diameter ca. 4 mm; sepals triangular, ca. 2 mm; stamens involute by petals, ca. 2 mm in length
**FRUIT**				
Size of fleshy fruit	8.5–10.2 × 8.2–10.1 mm	10.2–12.1 × 10.1–12.5 mm	9.7–11.1 × 8.9–10.1 mm	8.7–10.9 × 7.5–10.6 mm
**Size of dried fruit**	**6.0–7.0 × 4.7–5.3 mm**	**8.2–11.1 × 4.2–5.8 mm**	**7.5–9.8 × 4.5–5.5 mm**	**6.5–7.5 × 4.7–6.0 mm**
**Length-width ratio of dried fruit**	**1.3–1.5**	**1.6–2.2**	**1.6–2.0**	**1.3–1.5**
**Length of fruiting pedicel**	**5–9 mm**	**3–6 mm**	**5–9 mm**	**4–6 mm**
**SEED**				
**Size of seed**	**5.5–6.5 × 4.5–5.0 mm**	**7.1–9.9 × 4.0–5.5 mm**	**7.0–9.0 × 4.2–5.0 mm**	**5.0–7.0 × 4.5–5.5 mm**
**Length-width ratio**	**1.2–1.4**	**1.6**–**2.1**	**1.7**–**2.0**	**0.9**–**1.5**
**Seed apex**	**Rarely mucronate**	**Rarely mucronate**	**Mucronate or not**	**Mucronate**

**Table 3.** Nuclear ITS sequences variations between the two closely related *Rhamnella* species (*R. intermedia* vs. *R. rubrinervis*). The fixed nucleotides of *R. intermedia* are shown in bold. Variable positions interpreted based on the aligned sequences where mutation sites occur.

	Variable positions						
Species (individuals)	4	6	1	1	2	2	2
	2	9	1	2	1	2	2
			6	2	3	0	7
*R. intermedia* Type1 (10)	G	G	T	**T**	**T**	**T**	**C**
*R. intermedia* Type2 (5)	G	–	C	**T**	**T**	**T**	**C**
*R. intermedia* Type3 (1)	R	–	T	**T**	**T**	**T**	**C**
*R. rubrinervis* Type1 (5)	G	–	C	C	C	G	A
*R. rubrinervis* Type2 (16)	G	–	C	C	C	G	A

R: A/G.

### Taxonomic treatment

#### Rhamnella intermedia

Taxon classificationPlantaeRosalesRhamnaceae

Z. Qiang Lu & Y. Shuai Sun sp. nov.

A9CCB9AD-585B-597C-8261-0C67BFC92F4F

urn:lsid:ipni.org:names:77211387-1

Figures 1 , 2

##### Diagnosis.

*Rhamnella intermedia* differs from *R. rubrinervis* and *R. tonkinensis* by smaller size of dried fruit (6.0–7.0 × 4.7–5.3 vs. 7.5–11.1 × 4.2–5.8 mm) and seeds (5.5–6.5 × 4.5–5.0 vs. 7.0–9.9 × 4.2–5.5 mm) and smaller ratio of length to width of dried fruit (1.3–1.5 vs. 1.6–2.2) and seeds (1.2–1.4 vs. 1.6–2.1), and from *R. brachycarpa* by the larger ratio of length to width of leaves (2.7–3.8 vs. 1.9–2.4), smaller length of leaf petioles (3–8 vs. 7–12 mm), larger length of fruiting pedicels (5–9 vs. 4–6 mm), leaf apices acuminate to long acuminate and seed apices rarely mucronate. In addition, the characters of densely pilose young branches can also significantly separate this new species from *R. rubrinervis*.

##### Type.

China. Guangxi: Pingxiang County, 22°07'19"N, 106°44'40"E, 298 m altitude, karst limestone hill, 5 Oct 2019, *Z.Q. Lu 2019YG2601* (holotype, GXMI; isotypes, HITBC).

##### Description.

Shrubs or small trees, rarely climbing vines, evergreen. Young branches densely pilose; older branches grey-brown or grey, sparsely pilose or glabrous. Leaves alternate; stipules subulate, persistent; petiole 0.3–0.8 cm long, densely pilose when young, late sparsely pilose, rarely glabrous, narrowly grooved on the upper surface; leaf blade abaxially dark green, shiny, adaxially pale green, oblong or ovate-oblong, 6.4–13.0 × 2.0–5.0 cm, length-width ratio 2.7–3.8, leathery, abaxially sparsely pilose or glabrous, sparsely pilose along leaf veins or glabrous, adaxially glabrous, lateral veins 6–7 pairs, slightly impressed abaxially, prominent adaxially, base commonly rounded, rarely cuneate, margin inconspicuously remotely serrate or subentire; apex acuminate to long acuminate. Flowering branches axillary 8–13 cm long, densely or sparsely pilose, rarely glabrous. Flowers bisexual, ca. 4 mm diam., 5–merous, few to 10 in axillary cymes, cymes subsessile or shortly pedunculate at bracteole leaf of flowering branches; bracteole leaf similar to leaves in vegetative branches, but smaller, 3.5–6.5 × 1.3–2.0 cm, lateral veins 3–5 pairs. Pedicel 4.0–7.0 mm long, densely or sparsely pilose. Sepals triangular, ca. 2 mm, adaxially midvein raised, rostellate at lower middle. Petals obovate, shortly clawed. Stamens involute by petals, ca. 2 mm long. Disc rounded, thick. Ovary globose, not immersed in disc. Drupe purple-red or orange at maturity, ovoid-cylindrical or globose, 8.5–10.2 × 8.2–10.1 mm, 6.0–7.0 × 4.7–5.3 mm when dried, base with persistent calyx tube; fruiting pedicel 5.0–9.0 mm, sparsely pilose, 1-loculed, 1-seeded; seed short, apex rarely mucronate, smooth on the surface, 5.5–6.5 × 4.5–5.0 mm, length-width ratio 1.2–1.4.

##### Etymology.

Owing to its intermediate morphology compared to the other three closely related species, we provide the epithet *intermedia*.

##### Phenology.

Flowering from June to September and fruiting from August to October.

##### Habitat and distribution.

To date, only two *R. intermedia* populations have been collected from southwest Guangxi. For its population census, more than 20 mature trees (3–6 m in height) and a large number of seedlings grow on the karst limestone hill. We also found that six individuals present a climbing habit at locations where there is a relative high canopy; however, all others are erect. Interestingly, they shared the same ITS types between erect and climbing trees, suggesting no genetic differentiation. In addition, this new species may be also distributed in Vietnam, because it is found in Pingxiang and Daxin from southwest Guangxi, which is close to Vietnam. Further field investigations on its entire distribution are recommended in the future.

##### Additional specimens examined.

**China. Guangxi**: Pingxiang County, 22°07'19"N, 106°44'40"E, 298 m altitude, karst limestone hill, 5 Oct 2019, *Z.Q. Lu 2019YG2602*–*Z.Q. Lu 2019YG2619* (HITBC); Daxin County, Wude Township, 22°34'15"N, 106°44'56"E, 276 m altitude, along road, 27 May 2018, *Z.Q. Lu 201810801* (HITBC); the same locality: 26 August 2018, *Z.Q. Lu 201810802* (HITBC). Longzhou County, Daqingshan, hillside, July 1953, *Guangxi team 2967* (PE).

##### Notes.

*R. intermedia* is morphologically similar to *R. rubrinervis* based on leaf characters, but they can be easily distinguished between each other by fruit and seed characters. However, it also can be significantly distinguished from *R. rubrinervis* by the densely pilose young branches, if the specimen has no fruit and seeds. In addition, it also needs to be mentioned that the flower of *R. brachycarpa* is 5-merous.

### Key to four closely related evergreen *Rhamnella* species

**Table:** 

1	Dried fruit size 6.0–7.5 × 4.7–6.0 mm, length-width ratio 1.3–1.5; seed size 5.0–7.0 × 4.5–5.5 mm, length-width ratio 0.9–1.5	**2**
–	Dried fruit size 7.5–11.1 × 4.2–5.8 mm, length-width ratio 1.6–2.2; seed size 7.0–9.9 × 4.0–5.5 mm, length-width ratio 1.6–2.1	**3**
2	Young branch sparsely pilose or glabrous, leaf blade length-width ratio 1.9–2.4, apex short acuminate or acute, petiole 7–12 mm; fruiting pedicel 4–6 mm; seed apex mucronate	***R. brachycarpa***
–	Young branch densely pilose, leaf blade length-width ratio 2.7–3.8, apex acuminate to long acuminate, petiole 3–8 mm; fruiting pedicel 5–9 mm; seed apex rarely mucronate	***R. intermedia***
3	Leaf blade length-width ratio 2.1–2.8, petiole 7–11 mm; fruiting pedicel 5–9 mm	***R. tonkinensis***
–	Leaf blade length-width ratio 2.7–4.3, petiole 3–8 mm; fruiting pedicel 3–6 mm	***R. rubrinervis***

## Discussion

Many differentiated phenotypic traits between the evergreen *Rhamnella* species have been demonstrated (Lu and Sun 2019), such as length of leaf petioles, ratio of length to width of leaves, leaf apices and size and ratio of length to width of dried fruit and seeds. In the present study, we proposed two Guangxi populations as a new evergreen species, based on the following evidence. First, they are ascribed to the evergreen group that are significantly different from those deciduous broad-leaved species within *Rhamnella*, at the species level (Fan and Yang 1997; Lu and Sun 2019). Other characters such as drupe and seed sizes can also separate these two groups (Table 2; Chen and Schirarend 2007; Lu and Sun 2019). Second, they have intermediate morphology and stable phenotypic differences, and could be easily distinguished from the three closely related evergreen species within *Rhamnella* (Table 2). Third, PCA analysis further supported that they represent a distinct phenotypic cluster different from all published relatives (Figure 3; Lu and Sun 2019). However, the intermediate morphology of these two Guangxi populations also conforms to the characteristic of hybrids (Mallet 2005), which usually co-occur with parental species (Zha et al. 2010). Nevertheless, our field investigations show that none of the three closely related species co-occurred with this assumed new species. Furthermore, phylogenetic analysis of nuclear ITS sequence variations suggested they represented a distinct genetic lineage and closest to *R. rubrinervis* (Figure 4). Four fixed nucleotide sites were found between these two Guangxi populations and *R. rubrinervis* in the present study. Therefore, they are not hybrids, but represent an independent evolutionary lineage sister to *R. rubrinervis*. This new evolutionary lineage is distinguished from *R. rubrinervis* by densely pilose young branches, smaller size of dried fruit and seeds, and smaller ratio of length to width of dried fruit and seeds (Figure 1; Chen and Schirarend 2007). In total, these two Guangxi populations are distinctly different from all the published relatives. Hence, the two Guangxi populations are proposed to be erected as a new species, named as *R. intermedia*.

## Supplementary Material

XML Treatment for Rhamnella intermedia
